# Control of ticks and tick-borne diseases in Africa through improved diagnosis and utilisation of data on acaricide resistance

**DOI:** 10.1186/s13071-023-05803-3

**Published:** 2023-07-06

**Authors:** Richard P. Bishop, Naftaly W. Githaka, Thomas Bazarusanga, Chandra Bhushan, Abel Biguezoton, Patrick Vudriko, Dennis Muhanguzi, Maria Tumwebaze, Timbiira John Bosco, Caryn Shacklock, Josphat Kiama, Maxime Madder, Christine Maritz-Olivier, Weining Zhao, Francois Maree, Ayodele O. Majekodunmi, Lenaig Halos, Frans Jongejan, Alec Evans

**Affiliations:** 1 Washington State University, Pullman, WA USA; 2 International Livestock Research Institute (ILRI), Nairobi, Kenya; 3 Farmers Solutions Ltd., Kigali, Rwanda; 4 Elanco Animal Health, Monheim am Rhein, Germany; 5 CIRDES, Bobo Dioulasso, Burkina Faso; 6 Research Centre for Tropical Diseases and Vector Control (RTC) Makerere University, Kampala, Uganda; 7 Molecular and Computational Biology Research Group, Makerere University, Kampala, Uganda; 8 Veterinary Office Kiboga District, Kiboga, Uganda; 9 Afrivet Tick Unit, Howick, South Africa; 10 Department of Veterinary Services, Kabete, Kenya; 11 Clinglobal, Tamarin, Mauritius; 12 Department of Biochemistry, Genetics and Microbiology, University of Pretoria, Pretoria, South Africa; 13 United Nations Food and Agriculture Organisation, Rome, Italy; 14 Clinomics, Bloemfontein, South Africa; 15 Emergency Centre for Transboundary Diseases (ECTAD), Food and Agriculture Organisation of the United Nations, Rome, Italy; 16 Bill and Melinda Gates Foundation, Seattle, WA USA

**Keywords:** Acaricide resistance, Larval Packet Test (LPT), Ticks, *Rhipicephalus microplus*, *Rhipicephalus appendiculatus*, *Amidines (Amitraz), Synthetic pyrethroids, Organophosphates*

## Abstract

A meeting, sponsored by the Bill and Melinda Gates Foundation (BMGF) and organised by Clinglobal, was held at The International Livestock Research Institute (ILRI) in Nairobi, Kenya, from 19th – to 21st October 2022. The meeting assembled a unique group of experts on tick control in Africa. Academia, international agencies (FAO and ILRI), the private Animal Health sector and government veterinary services were represented. The significant outcomes included: (i) a shared commitment to standardisation and improvement of acaricide resistance bioassay protocols, particularly the widely used larval packet test (LPT); (ii) development of novel molecular assays for detecting acaricide resistance; (3) creation of platforms for disseminating acaricide resistance data to farmers, veterinary service providers and veterinary authorities to enable more rational evidence-based control of livestock ticks. Implementation of enhanced control will be facilitated by several recently established networks focused on control of parasites in Africa and globally, whose activities were presented at the meeting. These include a newly launched community of practice on management of livestock ticks, coordinated by FAO, an African module of the World Association for the Advancement of Veterinary Parasitology (WAAVP-AN) and the MAHABA (Managing Animal Health and Acaricides for a Better Africa) initiative of Elanco Animal Health.

**Graphical Abstract:**

**Figure:**
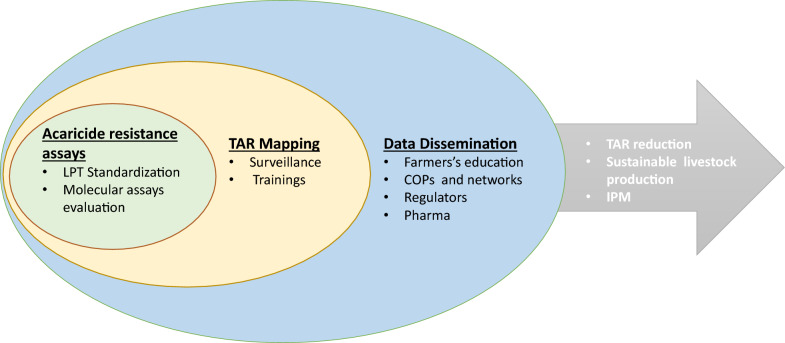

## The urgent requirement for improved tick control in sub-Saharan Africa

Ticks and tick-borne diseases significantly constrain livestock production globally, especially in Africa [1]. The economic and social impact of livestock ticks on resource-poor livestock keepers in Africa is substantial [2].

In contrast with other regions where *Rhipicephalus microplus* is the most important ixodid tick species causing mortality or morbidity in livestock, multiple tick species transmit additional major livestock pathogens through sub-Saharan Africa. These include East Coast fever (ECF), caused by the protozoan parasite *Theileria parva*, which is transmitted by the tick *Rhipicephalus appendiculatus*, and heartwater, caused by the rickettsial pathogen *Ehrlichia ruminantium* and transmitted by several species of ticks in the genus *Amblyomma* [1].

Ticks and tick-borne diseases in Africa have become increasingly severe because of the introduction of the invasive one-host tick, *R. microplus*, originating from Southeast Asia.

*Rhipicephalus microplus* is now well established in tropical and subtropical regions, including Latin America, Australia and, more recently, Africa. The economic importance of this tick species results from its capacity to transmit the highly pathogenic protozoans *Babesia bovis* and *B. bigemina* and the rickettsial pathogen *Anaplasma marginale*. After introduction of *R. microplus* to Madagascar in 1959, the tick spread to South Africa and Tanzania, frequently displacing the indigenous ‘blue tick’ *Rhipicephalus decoloratus*, notably in Tanzania [3]. More recently, *R. microplus* was introduced twice to West Africa (Benin and Cote D’Ivoire) from Brazil [4, 5] and has spread rapidly as far east as Cameroon, almost completely displacing the indigenous *R. decoloratus* in that country, [6] and possibly also the less frequently observed *Rhipicephalus annulatus* and *R. geigyi* (unpublished data). Data from Kenya similarly suggest *R*. *microplus* has displaced *R. decoloratus* from the coastal region [7].

Given the absence of alternative control measures, the application of acaricides for tick and tick-borne disease control remains the main practical option. Several recent publications have reviewed the status of acaricide deployment and resistance in Africa together with the perceptions of livestock keepers regarding ticks, tick-transmitted diseases and control methods [8–11].

However, as in other continents, resistance to the major classes of acaricides, synthetic pyrethroids, organophosphates, amidines and macrocyclic lactones [12, 13] has evolved rapidly in Africa. More data are required to clarify the extent of resistance and manage this in the field. It is crucial to distinguish resistance from misuse of chemical acaricides. The most recent guidelines on resistance management and parasite control in ruminants were published by FAO in 2004 [14] and highlighted the need for novel approaches for monitoring resistance suitable for resource-poor settings. These are urgently needed to support resistance mapping and inform vector management decisions and policies.

The goals of the meeting were to review the current knowledge relating to the prevalence of acaricide resistance in sub-Saharan Africa; learn from the progress of ongoing tick control initiatives; review updated protocols for standardisation of existing acaricide resistance diagnostics and assess the potential value of novel assays. There was also a discussion on enhancing the dissemination of accrued knowledge to farmers and national veterinary institute staff through digital and direct platforms for engagement with multiple clients (policymakers, DVS and DVO and farmers).

## Overview of the current status of acaricide resistance mitigation in East and West Africa

The keynote presentation was given by Dr Patrick Vudriko, Director of the RTC at Makerere University in Kampala, Uganda. He emphasised issues constraining efficient tick control and detecting acaricide resistance in Uganda and other countries in the region. His major emphasis was on the mitigation of resistance to *R. appendiculatus*, which transmits *T. parva* (causing East Coast fever in cattle), and the recently introduced *R. microplus*, transmitting highly pathogenic *B. bovis* and *A. marginale* to livestock, particularly cattle. He reviewed the current techniques for acaricide resistance diagnosis, in particular the larval packet test (LPT) and adult immersion test (AIT). These are currently relatively expensive and require a network of laboratories with the expertise and resources to perform them. Doctor Vudriko further highlighted additional dimensions of the acaricide resistance problem, including lack of resources, appropriate infrastructure and government extension services, leading to the suboptimal application of acaricides, and even crop pesticides instead of acaricides. Maria Tumwebaze, also of the RTC unit, presented data generated using the LPT and adult immersion test (AIT), indicating that resistance occurs among all three major classes of acaricides that are currently widely marketed in Uganda: synthetic pyrethroids, organophosphates and amidines.

Thomas Bazarusanga of ACRE Africa confirmed that resistance, as indicated using LPT, is also present against multiple acaracide types in Rwanda, with Amitraz being the most frequently implicated product (67–23% of cases, according to agroecological zone, with synthetic pyrethroids accounting for (1.6–1.7%). As in Uganda, suboptimal application of acaricides was observed as well as the use of unsuitable crop pesticides. Doctor Bazarusanga’s key recommendations for improvement of acaricide resistance surveillance (ARS) and the agencies with responsibility for this in Rwanda were summarised as enhanced extension services (RAB), enforcement of regulation at Rwanda Food and Drugs Authority (RFDA) and Licensing of Agrovet attendants from Rwanda Council of Veterinary Doctors (RCVD).

Abel Biguezoton of CIRDES summarised the current situation regarding ARS for eight countries in West Africa. In this region, the major tick species of economic importance are *Amblyomma variegatum* and *R. microplus*, and the main techniques used for assessment are LPT, AIT and larval immersion test (LIT). Maps were presented showing the geographical distribution of resistance within the region. For *R. microplus*, resistance in the field has been confirmed using the LPT in Burkina, Faso, Cote-D’Ivoire, Togo, Mali and Benin. As in Rwanda, Amitraz is the most frequently used product (68% of farms surveyed, with cypermethrin representing 13%). Unlike East Africa, acaricide application in West Africa is primarily by brushing, rather than spraying, using backpacks, which may result in differences in the evolution of acaricide resistance between East and West Africa.

Doctor Naftaly Githaka of ILRI provided a preliminary example of how diagnostic assays for acaricides could be integrated into the programmes of departments of veterinary services (DVS) in Africa through his work on acaricide-resistant *R. microplus* at the Kenyan coast. ILRI provides assay backstopping through quality control, training and, in future, the supply of reference susceptible tick strains. Trials of a similar collaboration between a dedicated centre of excellence, specifically the RTC at Makerere, and the District Veterinary Office of Kiboga, Uganda, were presented by Timbiira John Bosco, the regional DVO. Veterinarians were trained in tick collection, and subsequent analysis at RTC used the adult immersion technique (AIT).

## Bioassays for the diagnosis of acaricide resistance

The LPT remains the gold standard assay for evaluating acaricide resistance phenotypes in ticks. It has primarily been applied to diagnosing acaricide resistance to synthetic pyrethroids in *R. microplus* but can be adapted for organophosphates and amidines. Although the most widely adopted assay, it is a lengthy undertaking involving field sampling followed by six weeks in a specialised laboratory, because of the requirement to generate larval ticks from engorged adults. Professor Frans Jongejan of FAO summarised the history of the test and its application in Africa. The LPT was originally described and adopted in 1960s [15] and the current FAO guidelines for the administration of the test were published in 2004. Standardisation of the LPT was discussed, including the incorporation of susceptible reference ticks for each species evaluated (essential for data interpretation) and adaptation for more widespread use in low resource settings, through use of alternative materials and methodology. A presentation from Dr Caryn Shacklock of Afrivet, discussed alternative phenotypic assays, particularly the Shaw larval immersion test and their advantages and disadvantages. The potential of applying the larval tarsal test [16] as an alternative to LPT, allowing the testing of many compounds and doses in one multi-well plate, was also highlighted. This test avoids the relatively difficult handling of larvae required for LPT and has been validated in Brazil and Argentina. Wider evaluation in Africa would be worthwhile.

## Genetics of resistance and development of molecular assay

The two principal resistance mechanisms are metabolic resistance, associated with the enhancement of the detoxification enzyme system, or target-site resistance, associated with modification of the binding site of the acaricide. The tick proteins targeted by the most widely used acaricides are relatively well characterised. They are usually part of the nervous system and Single Nucleotide Polymorphism (SNP) at the target site have been correlated with resistance. The primary target sites for synthetic pyrethroids are the voltage-gated sodium channel; for organophosphates, the acetylcholinesterase receptor; and for macrocyclic lactones, the GABA-gated sodium channels. In the case of formamidines, which are Octopamine receptor agonists, the picture is the most complex, the octopamine/tyramine receptor contains SNPs implicated in resistance, but other genes, such as monoamine oxidases and ATP-binding cassette transporters, are also suspected targets. Professor Christine Maritz-Olivier presented the genetic complexity underpinning acaricide resistance phenotypes as shown in Table 1). It was emphasised that multiple SNPs within a gene, resulting in resistance phenotypes, and in the case of some acaricide classes, the existence of multiple target genes, will constrain the development of molecular assays that provide comprehensive information for all field situations. This may be particularly important in the case of formamidines (amitraz) since these are relatively cheap and typically the most widely used acaricides in Africa.

**Fig. 1:**
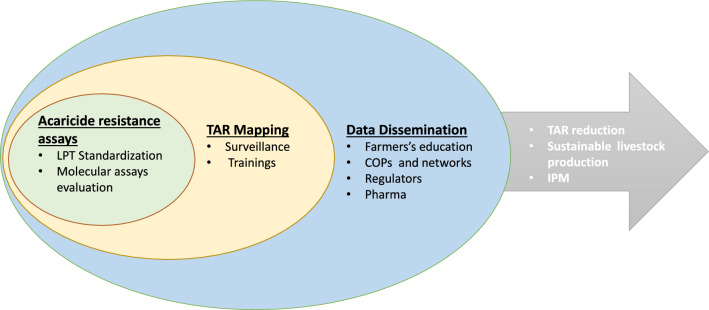
Acaricide resistance detection and mitigation strategy. The figure describes the major steps envisaged in the acaricide mitigation strategy. Improvement of existing bioassays and development of PCR-based molecular assays and training of users, surveillance of farming systems for resistant ticks using these tools, feedback of data collected to farmers and other stakeholders and more efficient use of acaricides leading to increased sustainability of livestock production

Based on sequence data derived from acaricide-resistant and susceptible *R. microplus* isolates from Africa and North and South America, Clinomics has developed a preliminary PCR-based assay for field detection of synthetic pyrethroid resistance in *R. microplus*. Dr Francois Maree presented the assay details and coordinated a practical demonstration for workshop participants. The current objective of the project is to show proof of concept for a field-applicable assay capable of providing rapid diagnosis of the presence of markers for acaricide resistance. The approach has the potential for multiplexing, incorporating additional targets for other acaricide classes in a format that may be used to support conventional bioassays in the future Fig. 1.

**Table 1:** Genetic basis of acaricide resistance phenotypes

Acaricide class	Active ingredients	Known target site(s)	Target site resistance (SNPs involved)	References
Pyrethroids	Cypermethrin Alphamethrin Flumethrin	Voltage-gated sodium channel Carboxylesterases	Domain II (L641 F72V) Domain III (F121) D374N	[17, 18] [19–21]
Cyclodienes/macrocyclic lactones	Dieldrin/Avermectins	GABA-gated chloride channel	T290L	[22]
Organophosphates	Chlorphenvinphos	Acetyl cholinesterase	Several SNPs in *BmAChEs*	[23, 24]
Formamidines	Amitraz	Octopamine/ Tyramine receptor B-andrenergic-like Octopamine receptor Monoamine oxidase Glutathione-S-transferase ATP-binding cassette transporters	T8P and L22S (OCT/TYR) 161R (*B*AOR)	[25–27] [28–30]

The figure highlights the currently known tick target genes involved in development of resistance to the four widely used classes of chemical acaricides: synthetic pyrethroids, macrocyclic lactones, organophosphates and formamidines. The nomenclature for specific SNPs known to confer resistance in particular genes is shown in column four

## Role of the private sector in creating solutions to address acaricide resistance

The MAHABA programme (Managing Animal Health and Acaricides for a Better Africa), managed by Elanco Animal Health and GALVmed and supported by BMGF, was presented by Dr Chandra Bhushan. The MAHABA programme is intended to deliver a practical strategy for managing ticks and the impact of tick-borne disease and to equip small-scale producers in Uganda and Nigeria with the necessary tools (acaricides) and knowledge (through a digital platform) to realise livestock productivity gains. This represents a significant advance relative to companies maximising acaricide sales without corresponding client engagement.

## Accelerating acaricide resistance mitigation through regional and global networks

Doctor Ayo Majekodunmi (WAAVP-AN co-chair) and Dr Weining Zhao (FAO) presented details on developing regional and global networks to support future research and guidelines for tick control. The WAAVP-AN is an African network of experts in veterinary parasitology. It is intended to improve knowledge on veterinary parasites as well as communication and networking on parasitology in Africa. Recently, FAO has established a global Community of Practice that will underpin the development of a new set of guidelines on acaricide resistance and the management of livestock ticks which can be accessed at (https://virtual-learning-center.fao.org/mod/page/view.php?id=7392).

## Future priorities for research and development

A key area will be the standardisation of protocols for phenotypic assays for acaracide resistance detection, with the LPT as an initial priority due to current widespread use. Some key issues for LPT include:

- Standardisation of the number of larvae used per packet and the number of replicates together with time scales for testing post hatching.
- Tick sampling strategy: ideally, a large number of ticks from multiple animals in a population should be collected to maximise the phenotypic diversity of ticks tested.
- Designation of specific central laboratories with the capacity and resources to maintain susceptible reference tick strains for distribution to regional testing hubs.
- Standardisation of interpretation of results between laboratories in order to improve consistency of the recommendations to farmers on optimal acaricide use.
- Promotion the use of alternative, cheaper substrates and solvents that will increase test affordability and application.
- These recommendations regarding LPT are presented in greater detail in Table 2.

**Table 2:** Larval packet test (LPT): key parameters and recommendations for standardisation of implementation in Africa

Larval packet test (LPT): parameters and recommendations for standardisation of implementation in Africa
Standardisation of the number of larvae used per packet (100 individuals is recommended) and the number of replicates. Also, time scales and conditions for testing including days post hatching and incubation conditions
Tick sampling strategy: As large a number as possible of ticks from multiple animals within a population should be collected to maximise the phenotypic diversity of ticks that are tested
Designation of specific central laboratories with the capacity and resources to maintain asset of universally recognised susceptible reference tick strains for distribution to regional testing hubs
Standardised interpretation of results between laboratories such as the use of the discriminating dose, LD_50_ or percentage mortality. This will strengthen the value of resulting recommendations to farmers on improvement of acaricide use
Experiment with alternative, cheaper substrates and solvents that will increase test affordability. For example explore use of locally available plant-based solvents instead of olive oil and trichloroethylene
Promote wider and more routine application of the LPT in labs throughout the African continent to diagnose resistance to all classes of chemical acaricide, including amidines and organophosphates, in addition to synthetic pyrethroids, which have been the major class of acaricides targeted. This may require use of alternative substrates such as nylon for Amitraz)
Agree on a set of dilutions of the active chemical ingredient for performance of the acaricide resistance assays

Additional priorities:

- Introduce the Larval Tarsal Test (LTT) to central laboratories and validate the results with LPT and future molecular tests. LTT could be a transition bioassay until the molecular tests are fully developed and validated.
- Improved data collection and centralised management to improve mapping and the dissemination of information to farmers, veterinary services and policymakers.
- Continued refinement of field-based molecular assays to increase the number of markers, acaricide classes and tick species which the assay can support.
- Evaluation of integrated control, including evidence-based implementation of acaricide rotation and combination strategies.
- Organisation of follow-up meetings with inclusive African country coverage, involving veterinary authorities and research organisations, to review resistance data in more detail and develop country-specific or regional models for implementation of acaricide resistance surveillance.

## Workshop conclusions

Acaricide resistance is widespread in Africa, and the impact on the control of ticks and tick-borne diseases has severe implications for farmer’s livelihoods. Multiple laboratories have confirmed the detection of resistance to all widely deployed classes of chemical acaricides. Furthermore, the problem is increasing because of the rapid spread of *R*. *microplus* within the continent and the reliance on acaricides to control this tick. This initial meeting of multiple experts highlighted the potential to deploy technical and knowledge-based solutions to mitigate the acaricide resistance problem in African livestock ticks and the role of recently implemented networks in catalysing this process.

## Data Availability

No detailed primary research data are included in this report. *Rhipicephalus microplus* acaricide susceptible reference ticks can be supplied to laboratories investigating acaricide resistance by Clinglobal.
